# Predictive role of high sensitivity troponin T within four hours from presentation of acute coronary syndrome in elderly patients

**DOI:** 10.1186/s12873-015-0064-z

**Published:** 2016-01-04

**Authors:** Catharina Borna, Katarina Lockman Frostred, Ulf Ekelund

**Affiliations:** Department of Clinical Sciences at Lund, Section of Emergency Medicine, Lund University, Lund, Sweden

**Keywords:** High sensitivity troponin T, Acute coronary syndrome, Elderly patients

## Abstract

**Background:**

Previous studies indicate that the introduction of high-sensitivity troponin T (HsTnT) as a diagnostic tool for chest pain patients in the emergency department (ED) creates a high rate of false-positive tests. In the present study, we aimed to evaluate if the diagnostic performance of HsTnT for acute coronary syndrome (ACS) up to 3–4 h after presentation in elderly patients can be improved.

**Methods:**

A total of 477 consecutive patients ≥ 75 years, admitted to in-hospital care for chest pain suspicious of ACS, were retrospectively included. HsTnT values at presentation (0 h) and at 3–4 h were analysed. Receiver operating characteristic (ROC) curves were created for absolute and relative changes from 0 to 3–4 h. ACS, non-elective percutaneous coronary intervention, coronary artery bypass grafting and death of all causes were recorded for all patients during a follow-up of 60 days. Sensitivity, specificity, negative predictive value (NPV) and positive predictive value (PPV) were analysed for different HsTnT cut-off values at 0 and 3–4 h and for the combination of a HsTnT at presentation and an absolute change from 0 to 3–4 h.

**Results:**

Twenty-seven percent of the patients had ACS and 21 % acute myocardial infarction (AMI) during the hospital stay. The standard cut-off 14 ng/L gave sensitivity and NPV for ACS of 88 and 90 % at 3–4 h. Specificity and PPV was 38 and 32 % respectively. Analysing for non-ST elevation myocardial infarction (NSTEMI) alone gave a sensitivity and NPV of 100 % but did not improve specificity and PPV. The area under the ROC-curve was larger for absolute than relative HsTnT changes from 0 to 3–4 h. A combination of HsTnT at presentation > 30 ng/L and/or a change > 5 ng/L up to 3–4 h gave a 63 % specificity and a PPV of 46 %, a 99 % sensitivity and a NPV of 99 % for NSTEMI.

**Conclusion:**

Our study indicates that HsTnT can neither exclude nor confirm ACS within 3–4 h from presentation in patients ≥ 75 years. NSTEMI can be excluded with HsTnT within 3–4 h, but HsTnT cannot be used to rule in NSTEMI during the first 3–4 h, not even by using a combination of the initial HsTnT result and the change from 0 to 3–4 h. With combined criteria, the majority of the positive tests were still false positive. Our results indicate that in patients > 75 years, HsTnT should be used primarily as an early rule-out tool for AMI.

## Background

The cornerstones in the evaluation of emergency department (ED) patients with possible acute coronary syndrome (ACS), including acute myocardial infarction (AMI), are the ECG, the symptom history, and blood markers of myocardial necrosis such as troponin. With the introduction of high-sensitivity assays, extremely low concentrations of cardiac troponins in the blood can be measured with excellent precision [1]. However, increased analytic sensitivity of troponin assays not only increases detection of AMI [2, 3], but also of the many other conditions with elevated troponin levels [4, 5]. These conditions, e.g. pulmonary embolism, heart failure, tachycardia and renal failure are more common in elderly patients.

In the latest European guidelines [6], the AMI diagnosis requires a rise or fall in blood troponin with at least one value above the 99th percentile [7]. It is also stated that ACS may be excluded with a rapid 3 h high-sensitivity troponin sampling protocol.

With the high-sensitivity troponin T (HsTnT) assay the level of the 99th percentile is as low as 14 ng/L. Previous studies indicate that this cut-off creates a high rate of false-positive tests [8], and concerns have been raised that this decreases the diagnostic value of HsTnT for AMI in elderly patients [8–13].

In the present study, we aimed to evaluate if the diagnostic performance of HsTnT for ACS up to 3–4 h after presentation in elderly patients could be improved.

## Methods

### Study site

The Skåne University Hospital in Lund serves as the primary hospital for approximately 300 000 inhabitants. The hospital has a cardiac intensive care unit with 19 beds and an observation unit with ECG monitoring at 20 beds. Percutaneous coronary intervention (PCI) and coronary artery bypass grafting (CABG) are available 24 h a day. Some 65 000 patients present to the ED per year, and approximately 5500 of these present with acute chest pain. There is no standardized diagnostic protocol for patients with suspected ACS, and no dedicated chest pain unit. A prehospital ECG system is in operation, with ambulance ECGs sent to a cardiologist on call. If an ST elevation myocardial infarction (STEMI) is identified, the patient is transported directly to the angiography laboratory, bypassing the ED.

### Inclusion of patients

All patients ≥ 75 years with chest pain suspicious of ACS, as identified and noted in the patient records by the responsible ED physician, were retrospectively included in the study database if they were admitted from the ED to the cardiac intensive care unit or the medical observation unit during the period of February to April 2010, March to July 2011 and October 2011 to March 2012. The study period was divided into three for practical reasons and due to the limitations in the hospital’s administrative routines. Patients identified as very low risk by the responsible ED physician were directly discharged home from the ED and were not included in the study. We also excluded patients with STEMI, cardiac arrest at the ED or living outside the region. The study was approved by the the Regional Ethical Review Board in Lund (http://www.epn.se), registry number 2010/429. The Regional Ethics Review Board did not request written informed consent.

### Clinical assessment

All patients underwent routine assessment in the ED including physical examination, 12-lead ECG and laboratory analyses including HsTnT. After admission to the observation or the cardiac intensive care unit, all included patients were subjected to continuous ECG monitoring, pulse oxymetry and non-invasive blood pressure measurements. Blood samples for HsTnT analysis were collected at presentation (0 h) and thereafter at the discretion of the attending physician, but mainly at 3–4 h and 6–7 h from presentation according to the ESC guidelines [6].

### High sensitivity troponin T assay

The HsTnT analyses were performed with the use of the Elecsys 2010 system (Roche Diagnostics) with a limit of detection of 2 ng/l, a 99th-percentile cut-off point of 14 ng/l, and a coefficient of variation of less than 10 at 13 ng/l.

### Recording of patient diagnoses and endpoints

For patients admitted to in-hospital care, the final discharge diagnosis (including the ICD10 code) based on clinical data from the entire hospital stay was retrieved from the discharge summary written by the ward physician and reviewed for quality by the responsible specialist ward physician (cardiologist and/or internal medicine specialist). The diagnostic criteria for ACS, AMI or UA used at the hospital during the study were those recommended by the European Society of Cardiology, the American College of Cardiology and the American Heart Association [7]. HsTnT values above 14 ng/L were considered indicative of ACS. In addition, all diagnoses and ECGs were reviewed by two of the authors (CB, specialist in cardiology and internal medicine, and KLF, specialist in internal and emergency medicine) who finally decided each diagnose in consensus using all available clinical data for each patient. In patients with an HsTnT > 14 ng/L, a 20 % rise or fall was considered sufficient for an AMI diagnosis together with a clinical course suggestive of ACS, i.e. typical pain location and duration, ECG changes and no other obvious cause of the chest pain. [14]. The UA diagnosis was considered correct when a patient had an HsTnT that was normal or without dynamic changes, together with deterioration of previous stable angina, typical chest pain at rest, or had a positive cardiac stress test or significant stenosis on coronary angiography. A type 2 AMI was deemed by the reviewers to be present when there was ischemic myocardial damage due to imbalance between oxygen demand and supply, as in e.g. tachycardia or hypoxemia. Since we focused on the diagnostic value of HsTnT for downstream ACS management, these patients were considered as non-ACS cases in all analyses except for the group called “all AMI”, see Table 2.

The endpoints analysed were ACS, NSTEMI, all AMI (NSTEMI and Type 2 AMI) and MACE (NSTEMI, non-elective PCI, non-elective CABG and death of all causes within 2 months, including the initial stay).

### Statistical analysis

#### Patient characteristics

Continuous variables are presented as medians (with inter-quartile range) and were compared with the Mann–Whitney test. Categorical variables were compared with the Pearson Chi-square test.

#### Assessment of diagnostic performance

Sensitivity, specificity, negative predictive value (NPV) and positive predictive value (PPV) were analysed for HsTnT cut-off values at 3–4 h and for the combination of a cut-off value at presentation and an absolute change from 0 to 3–4 h. The different cut-off values were pre-specified and based on previous studies [11, 12]. Sensitivity and specificity with 95 % confidence intervals were calculated. A sensitivity of ≥ 98 % was considered as a clinically acceptable rule out level [15].

Receiver operating characteristic (ROC) curves were created and the areas under the curves (AUROC) compared for absolute and relative HsTnT changes (in percent) from presentation to 3–4 h. IBM SPSS Statistics 18 (New York, USA) was used for statistical analyses, and a *p* < 0.05 was regarded as statistically significant.

For calculations of 95 % confidence intervals (CI) VassarStats clinical calculator (Vassar College, Poughkeepsie, NY: http://faculty.vassar.edu/lowry/clin1.html) was used.

## Results

### Patient outcomes and characteristics

A total of 477 patients with suspected ACS were included in the study. As seen in Table 1, 127 patients (27 %) had a final discharge diagnosis of ACS and of these, 28 had UA. Nineteen of the UA patients (68 %) were diagnosed after coronary angiography. Twenty-five patients (5 %) were considered to have type 2 AMI, and these are included in the non-ACS group. In total, 143 patients (30 %) had a MACE within 2 months. Twelve patients died after the initial hospital stay and six patients were diagnosed with ACS after the initial stay but within 2 months. None of the patients diagnosed with ACS after the initial stay died. The causes of death were not registered.

**Table 1:** Characteristics and outcomes for study patients

		All	ACS	Non-ACS	*p value*
		(*n* = 477)	(*n* = 127)	(*n* = 350)	*(ACS vs non-ACS)*
Age, median (IQR) years		82 (77–85)	82 (78–85)	81 (68–88)	0.38
Male sex		253 (53)	77 (61)	175 (50)	0.04
Medical history	Hypertension	279 (59)	79 (62)	200 (57)	0.33
	Hyperlipidemia and/or use of statins	230 (48)	63 (50)	167 (48)	0.72
	Diabetes	116 (24)	38 (30)	78 (22)	0.09
	CAD	281 (59)	74 (58)	207 (59)	0.86
	Prior PCI	130 (27)	34 (27)	96 (27)	0.89
	Prior CABG	94 (20)	23 (18)	71 (20)	0.60
	Stroke	86 (18)	24 (19)	62 (18)	0.77
	CHF	90 (19)	27 (21)	63 (18)	0.40
Smoking status	Current	32 (6.7)	10 (8.4)	21 (6.1)	0.70
Current medication	Warfarin	87 (18)	11 (9)	76 (22)	<0.01
Findings on presentation	Heart rate (beats/min)	77	80	76	0.08
	Systolic blood pressure (mmHg)	150	150	147	0.20
	Diastolic blood pressure (mmHg)	80	80	80	0.04
ECG	ST depression	53 (11)	32 (25)	21 (6)	<0.01
	ST elevation	13 (3)	5 (4)	8 (2)	0.33
	T-Wave inversion	21 (4)	9 (7)	12 (3)	0.09
	Left bundle branch block	7 (1)	3 (2)	4 (1)	0.33
	Right bundle branch block	3 (1)	0	3 (1)	0.30
	Non interpretable ECG (pacemaker)	44 (9)	14 (11)	30 (9)	0.41
Laboratory tests	CRP (mg/L)	2.9	2.8	2.9	0.73
	Creatinine (μmol/L)	94	99	92	0.04
	Cholesterol (mmol/L)	4.2	4.2	4.1	0.69
	Troponin at presentation median (IQR)	20 (11–37)	37 (19–116)	17 (8–28)	<0.01
	Troponin positive at presentation	297 (63)	100 (81)	197 (58)	<0.01
	Abs diff T0T3	2 (1–7)	18 (4–67)	2 (0–4)	<0.01
GRACE score median (IQR)		142 (125–164)	150 (128–177)	140 (124–156)	0.01
In-hospital outcome	Coronary angiography	104 (22)	83 (65)	21 (6)	<0.01
	PCI	39 (8)	39 (8)	0	<0.01
	CABG	27 (6)	27 (6)	0	<0.01
	Death	13 (3)	7 (6)	6 (2)	0.02

Data are given as n (%) or median (interquartile range [IQR]). *CAD* coronary artery disease, *CHF* congestive heart failure, *ACS* acute coronary syndrome, *PCI* percutaneous coronary intervention, *CABG* coronary artery bypass grafting, *GRACE* Global Registry of Acute Coronary Events, *Abs diff T0T3* absolute change in HsTnT from admission to 3 h. Standard clinical definitions of hypertension, Hyperlipidemia, Diabetes, CAD, stroke and CHF were used

Only 49 of the patients with ACS (38 %) presented with ECG changes not known to be old. As seen in Table 1, there were no significant differences in prior cardiovascular disease between patients with and without ACS. Male sex was significantly more common and warfarin treatment significantly less common in the ACS group. Average age was similar in patients with and without ACS, but GRACE score was significantly higher in the group with ACS. HsTnT levels and the number of HsTnT positive (>14 ng/L) patients at ED presentation were significantly higher in patients with ACS than in the non-ACS group.

### HsTnT analysis at presentation and at 3–4 h

With the cut-off 14 ng/L, the sensitivity for NSTEMI at presentation was 91 % (CI 95 % 83–91) with a specificity of 43 % (CI 95 % 38–48). Increasing the cut-off values at presentation resulted in even lower levels of sensitivity (data not shown).

Three hundred and twelve patients (65 %) had an hsTnT result at 3–4 h. In Table 2, panel a, it can be seen that with the cut-off 14 ng/L, the sensitivity for ACS was 88 % at 3–4 h, with a specificity of 38 %. Analysing for NSTEMI alone (panel b) gave a sensitivity and NPV of 100 % but did not improve specificity and PPV. When diagnostic performance for all AMI (NSTEMI and Type 2 AMI) were analysed (panel c), sensitivity and NPV reached 100 % but specificity remained low at 41 %. The sensitivity and specificity for MACE at 2 months (panel d) were 91 and 39 %. Using the cut-offs 20 and 30 ng/L decreased sensitivity and increased specificity for all endpoints.

**Table 2:** Diagnostic performance for ACS (a), NSTEMI (b), all AMI (c) and MACE after 2 months (d) of HsTnT analysed 3–4 h

a) ACS prediction at 3–4 h	Sensitivity	Specificity	NPV	PPV
*n* = 77	(95 % CI)	(95 % CI)	(95 % CI)	(95 % CI)
HsTnT				
>14	88 (78–94)	38 (32–44)	90 (83–95)	32 (26–35)
>20	81 (70–88)	58 (52–65)	90 (84–90)	39 (31–47)
>30	77 (65–85)	75 (69–80)	91 (86–94)	50 (41–59)
b) NSTEMI prediction at 3–4 h				
*n* = 61				
HsTnT				
>14	100 (93–100)	39 (33–45)	100 (95–100)	28 (22–35)
>20	93 (83–98)	59 (53–65)	97 (93–99	36 (28–44)
>30	90 (79–96)	75 (69–80)	97 (93–99)	47 (37–56)
c) All AMI prediction at 3–4 h				
*n* = 81				
HsTnT				
>14	100 (94–100)	41 (35–48)	100 (95–100)	36 (32–43)
>20	94 (85–98)	63 (56–69)	97 (92–99)	45 (37–53)
>30	90 (80–95)	80 (73–84)	96 (92–98)	58 (49–67)
d) MACE prediction at 3–4 h				
*n* = 86				
HsTnT				
>14	91 (83–96)	39 (33–46)	93 (85–97)	35 (29–42)
>20	83 (73–90)	60 (54–66)	91 (85–95)	42 (35–51)
>30	77 (66–85)	77 (70–81)	90 (85–94)	53 (44–63)

*TnT* high-sensitivity troponin T, *AMI* acute myocardial infarction, *NSTEMI* non-ST-elevation myocardial infarction

All AMI includes NSTEMI and Type 2 AMI. MACE, NSTEMI, non-elective PCI, non-elective CABG and death within 2 months

### HsTnT changes

**Fig. 1:**
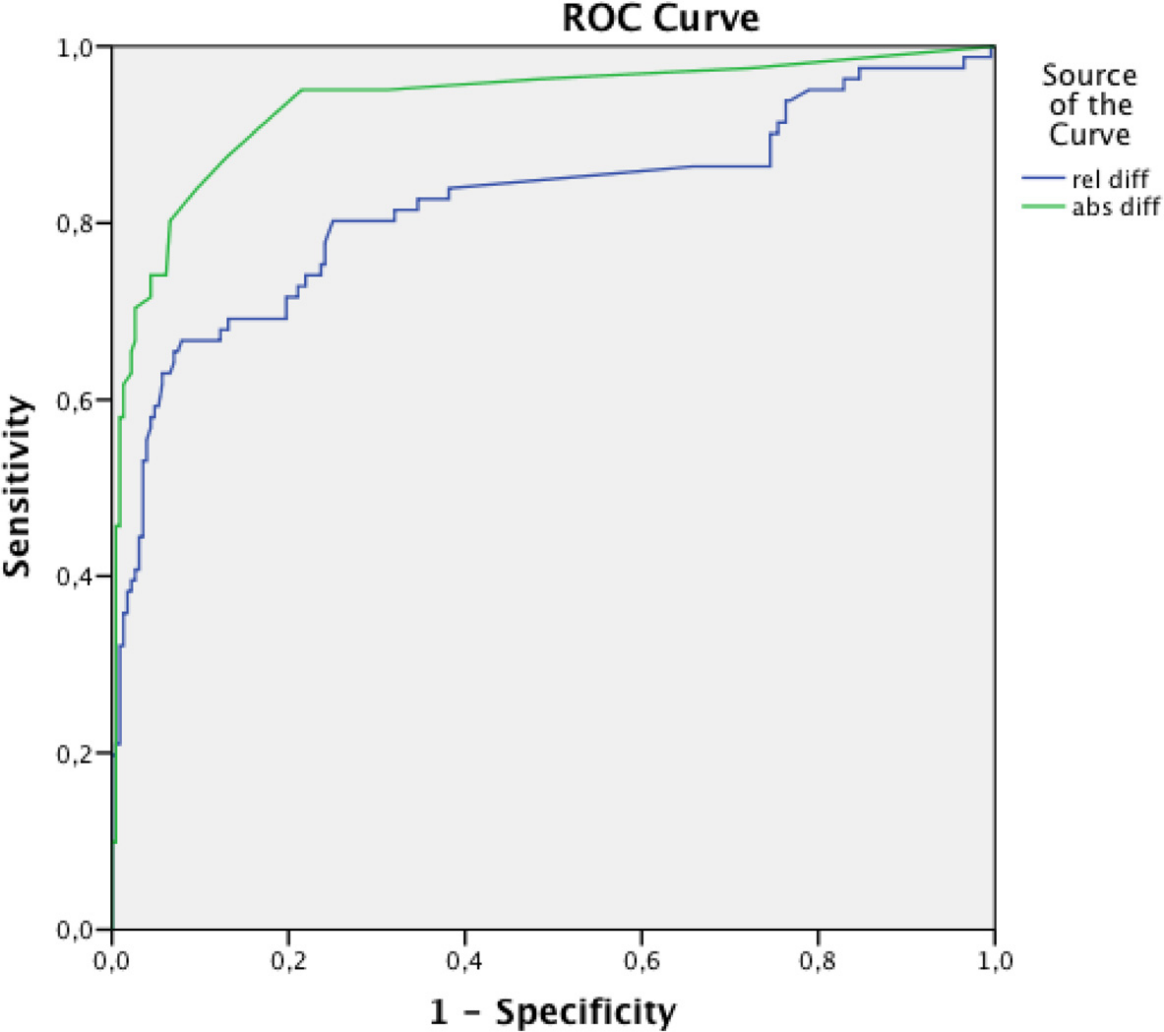
ROC curves for AMI prediction of absolute and relative HsTnT changes from presentation to 3–4 h. Areas under the curves: 0.94 (95 % confidence interval 0.899–0.972) for absolute HsTnT change and 0.82 (95 % confidence interval 0.759–0.885) for relative change

For the diagnosis of AMI, areas under the ROC curves for relative and absolute HsTnT changes were analysed. As shown in Fig. 1, AUROC was larger for absolute than for relative HsTnT changes from 0 to 3–4 h.

### Single HsTnT analyses combined with HsTnT changes

In an attempt to improve specificity and PPV for NSTEMI, a single HsTnT value at presentation was combined with the absolute HsTnT change from 0 to 3–4 h, and the results are shown in Table 3. A combination of HsTnT at presentation > 30 ng/L and/or a change > 5 ng/L gave a 63 % specificity and a PPV of 46 %, a 99 % sensitivity and a NPV of 99 % for NSTEMI. On the assumption that 98 % sensitivity is acceptable in routine care, these criteria would allow NSTEMI to be ruled out at 3–4 h in 47 % of the patients.

**Table 3:** Diagnostic performance for NSTEMI with analysis at single time points and/or changes

		Sensitivity (95 % CI)	Specificity (95 % CI)	NPV (95 % CI)	PPV (95 % CI)
HsTnT at presentation	>14				
and/or abs Δ to 3–4 h	>5	99 (93–100)	30 (25–36)	99 (94–100)	30 (25–35)
	>7	98 (92–100)	32 (27–38)	98 (92–100)	30 (25–36)
	>9	96 (89–99)	32 (27–38)	96 (90–99)	30 (25–35)
HsTnT at presentation	>20				
and/or abs Δ to 3–4 h	>5	99 (93–100)	46 (41–52)	99 (96–100)	36 (30–43)
	>7	98 (92–100)	49 (43–54)	99 (95–100)	37 (31–44)
	>9	96 (89–99)	49 (43–54)	97 (93–99)	37 (31–43)
HsTnT at presentation	>30				
and/or abs Δ to 3–4 h	>5	99 (93–100)	63 (57–68)	99 (96–100)	46 (40–54)
	>7	97 (90–99)	66 (60–72)	98 (95–100)	48 (41–56)
	>9	94 (87–98)	67 (61–72)	97 (94–99)	48 (41–56)
HsTnT at presentation	>40				
and/or abs Δ to 3–4 h	>5	95 (88–98)	70 (64–76)	98 (94–99)	51 (43–59)
	>7	91 (82–96)	75 (70–80)	96 (92–98)	54 (46–62)
	>9	88 (80–94)	76 (70–81)	95 (91–98)	54 (46–63)

*TnT* high-sensitivity troponin T, *NSTEMI* non-ST-elevation myocardial infarction, abs Δ to 3–4 h, absolute change TnT from admission to 3–4 h. A sensitivity of ≥ 98 % is considered acceptable for rule out

Best diagnostic performance is marked with grey

Increasing the cut-offs for the initial HsTnT or the absolute change decreased the sensitivity to less than 98 %. A combination of the HsTnT result at 3–4 h with the change from 0 to 3–4 h did not improve these results (data not shown).

### Patients without ACS

Of 477 study patients, 350 (73 %) did not have ACS during the index visit. HsTnT at presentation was pathological in 197 (58 %) of these patients (Table 1). Table 4 shows the discharge diagnoses for all patients. As can be seen, an elevated HsTnT at the ED was very common in patients with pneumonia, pulmonary embolism and heart failure. All patients with type 2 AMI had clinical conditions that made in-hospital care necessary regardless of the HsTnT value.

**Table 4:** Discharge diagnoses (ICD10, all patients)

	ICD10	Number	Percent	% hsTnT > 14 at presentation	% hsTnT > 30 at presentation
Ischaemic heart disease	I20-I25	161	34	74	51
Chest pain	R07	135	28	42	15
Atrial fibrillation and flutter	I48	36	8	78	19
Heart failure	I50	14	3	86	43
Pneumonia	J18	11	2	100	46
Non specified disease	Z00	9	2	67	33
Soft tissue disorder	M79	7	2	42	14
Pulmonary embolism	I26	6	1	100	33
Gastro-esophageal reflux disease	K21 + K29	6	1	17	17
Other diagnoses	-	92	19	-	-

## Discussion

In the present study in patients > 75 years, there were three main findings. *First*, the rapid 3 h HsTnT protocol suggested by the latest European guidelines cannot be used to rule in or rule out ACS during the hospital stay or MACE within two months. *Second*, a single HsTnT at 3–4 h from patient presentation can rule out NSTEMI. *Third*, HsTnT cannot be used to rule in NSTEMI during the initial 3–4 h using either standard or adjusted cut-offs, or a combination of the initial HsTnT result and the change from 0 to 3–4 h.

In patients with possible ACS, the main diagnostic methods in the ED are symptom history, ECG and blood tests such as HsTnT. The chest pain history was not recorded in our study, but it is well known that elderly ACS patients often have atypical symptoms such as shortness of breath or fatigue, which makes the diagnosis difficult [16]. The diagnostic yield of the ECG was low in our patient population, since the ECG was normal or unchanged from a previous ECG in 62 % of the ACS patients. In view of this, it seems important to optimize the diagnostic performance of HsTnT in elderly patients in order to avoid unnecessary admission to in-hospital care, over-investigation and over-treatment with anticoagulant and antiplatelet drugs for ACS, especially since elderly patients have more comorbidity than younger patients and are at a higher risk of adverse drug effects.

The European Society of Cardiology (ESC) state that non-ST-elevation acute coronary syndromes can be excluded with a rapid 3 h HsTn sampling protocol [17]. We found that this protocol, using the standard cut-off 14 ng/L, had a sensitivity for ACS at 3–4 h of less than 90 % with a NPV of 90 %. Our conclusion is that the ESC’s rapid HsTn sampling protocol cannot rule out ACS within 3–4 h after presentation. These results are in accordance with our previous finding in patients of all ages that HsTnT cannot rule out ACS up to 6–7 h after presentation [8], and with the results of the meta-analysis by Sethi et al. [18].

However, in our patients > 75 years we observed that a single HsTnT at 3–4 h was an excellent way to exclude NSTEMI; the sensitivity was 100 %. This finding has been well described in previous studies [8, 19]. After NSTEMI has been excluded however, the question remains whether the patient can be safely discharged from the ED or whether he or she should be further assessed for UA. Since UA is a difficult and often ambiguous diagnosis, especially in elderly patients who often do not undergo coronary angiography due to the inherent risks, we also evaluated the diagnostic performance of HsTnT for MACE within 2 months. MACE included more robust events such as NSTEMI, non-elective PCI or CABG and death of all causes. Sensitivity and NPV for MACE were similar to those for ACS alone, indicating that excluding NSTEMI at the ED is not sufficient for optimal care, even if the 60-day follow-up in this study was quite generous.

This study indicates that HsTnT cannot be used to rule in NSTEMI during the initial 3–4 h using either standard or adjusted cut-offs, or a combination of the initial HsTnT result and the change from 0 to 3–4 h.

Using the standard cut off 14 ng/L, the ability of a single HsTnT at 3–4 h to rule in both NSTEMI and all AMI (NSTEMI and Type 2 AMI) was poor, since specificities and PPVs were below 50 %. This low specificity of HsTnT for AMI (and ACS) confirms previous results by Borna [8], Bahrmann [9] and Olivieri et al. [20], and is also well described in the meta-analysis by Sethi et al. [18]. Conditions associated with elevated troponin levels are frequent in the elderly, and in our study the initial HsTnT was > 14 ng/L in all patients with pneumonia or pulmonary embolism, and in almost all patients with heart failure or atrial fibrillation (Table 4). Taken together with the considerable biological variation in the HsTnT level over time [21], this can explain why the diagnostic yield of the HsTnT change during the initial hours for rule-in was limited in patients ≥ 75 years. Another possible cause is that some of the patients were so called “late presenters” and arrived to the ED with an established infarction. The time of chest pain onset was not registered in the present study.

Increasing the cut off values for single HsTnT tests (Table 2) improved specificity but at a clinically acceptable sensitivity of ≥ 98 %, PPV did not exceed 50 %. HsTnT as a single analysis in the elderly thus has similarities with the d-dimer test where a positive result is not useful to rule in thrombosis due to its low specificity and PPV [22].

In order to improve PPV and reduce the number of false positive tests, we evaluated a combination of a higher HsTnT cut-off at presentation and a low HsTnT change up to 3–4 h. In this way, patients without AMI but with an elevated baseline troponin level were not classified as AMI, while AMI patients with low baseline HsTnT values could still be identified by the HsTnT change. Like previous studies we found that absolute change in HsTnT was diagnostically superior to relative change [23, 24]. Our combined criteria considerably improved specificity and PPV, and with HsTnT at presentation > 30 ng/L and/or a change > 5 ng/L, NSTEMI could be ruled out within the first 3–4 h in 47 % of unselected ED patients > 75 years.

Combinations of adjusted cut-off values at presentation and HsTnT changes over time have been evaluated in two previous studies. Reichlin et al. [25] reported that a 12 ng/L cut-off at presentation and/or change in HsTnT of < 3 ng/L within the first hour ruled out AMI in 60 % of chest pain patients of all ages with a sensitivity and NPV of 100 %. Similarly, Bahrmann et al. [19] combined an HsTnT < 23 ng/L at presentation and/or a change < 3 ng/L within the first 3 h and could rule out NSTEMI in one-third of unselected ED patients older than 70 years.

Despite the improved specificity and PPV with our combined criteria, it seems unlikely that they will improve clinical care. With combined criteria, PPV still did not reach more than 46 % when aiming at a sensitivity of ≥ 98 %. More than half of our elderly patients with positive criteria did thus not have NSTEMI. Additional information and observation after 3–4 h will then be needed to make an NSTEMI diagnosis. Such a rule in process could last for many hours or even days, since the diagnosis will then depend on dynamic HsTnT changes during the most likely “non-dynamic” phase of troponin release from the infarcted myocardium [26]. Perhaps we will soon be ready to accept that it will often be impossible to assign a working diagnosis of AMI using only troponin as Rains et al. state in their review on biomarkers of AMI in the elderly [27].

### Limitations

This retrospective study was performed at one university hospital only and all patients were admitted to wards with capacity for close monitoring. Only patients who were hospitalized following admission from the ED were included. The results are not necessarily generalizable to other patient populations, and need to be confirmed in a prospective study.

As in many other studies on the diagnostic value of troponin, incorporation bias was a substantial weakness in our study. Although the discharge diagnoses were based on clinical information from the entire hospital stay, including HsTnT testing after 3–4 h, the HsTnT results in this study were part of the information used to make the discharge diagnosis. In addition, the review of the discharge diagnoses was not independent, but performed by the authors.

Another limitation in the interpretation of the results is the lack of a gold standard. There is no clear definition on what dynamic changes of HsTnT are necessary for the diagnosis of AMI. Further, consensus is lacking regarding the diagnostic criteria for UA. Although many were based on coronary angiography, the UA diagnoses in this study may therefore not be entirely reproducible.

In the present paper, patients who were deemed to have type 2 AMI were analyzed in the non-ACS group. It could be argued that type 2 AMI diagnoses should be included in the ACS group, but as it stands, troponin is primarily used to identify patients with acute coronary disease. A type 2 AMI diagnosis can be difficult to establish, especially in the absence of a coronary angiography, but these diagnoses were based on consensus among the 2 reviewers and on evidence of little or no coronary disease, e.g. previous stress tests. The type 2 AMI diagnoses should therefore be reasonably correct.

## Conclusion

Our study indicates that in patients ≥ 75 years, HsTnT can neither exclude nor confirm ACS within 3–4 h of presentation, but that a single HsTnT at 3–4 h can exclude NSTEMI. In contrast, HsTnT cannot be used to rule in NSTEMI during the first 3–4 h, not even by using a combination of the initial HsTnT and/or the change up to 3–4 h. With combined criteria, the majority of the positive tests were still false positive. HsTnT in the elderly is thus similar to the d-dimer test, where a positive result cannot rule in venous thrombo-embolism. Our results indicate that in patients > 75 years, HsTnT should be used primarily as an early rule-out tool for AMI.

## Abbreviations

ACS: acute coronary syndrome
AMI: acute myocardial infarction
AUROC: area under the curve
CABG: coronary artery bypass grafting
CI: confidence interval
ED: emergency department
HsTnT: high-sensitivity troponin T
MACE: major adverse cardiac event
NPV: negative predictive value
NSTEMI: non-ST elevation myocardial infarction
PPV: positive predictive value
PCI: percutaneous coronary intervention
ROC: receiver operating characteristic
STEMI: ST elevation myocardial infarction

## References

[1] Saenger AK, Beyrau R, Braun S, Cooray R, Dolci A, Freidank H (2011). Multicenter analytical evaluation of a high-sensitivity troponin T assay. Clin Chim Acta.

[2] Reichlin T, Hochholzer W, Bassetti S, Steuer S, Stelzig C, Hartwiger S (2009). Early diagnosis of myocardial infarction with sensitive cardiac troponin assays. N Engl J Med.

[3] Keller T, Zeller T, Peetz D, Tzikas S, Roth A, Czyz E (2009). Sensitive troponin I assay in early diagnosis of acute myocardial infarction. N Engl J Med.

[4] Agewall S, Giannitsis E, Jernberg T, Katus H (2011). Troponin elevation in coronary vs. non-coronary disease. Eur Heart J.

[5] Vuilleumier N, Limacher A, Mean M, Choffat J, Lescuyer P, Bounameaux H, et al. Cardiac biomarkers and clinical scores for risk stratification in elderly patients with non-high-risk pulmonary embolism. J Intern Med. 2014. Epub 2014/10/07. 10.1111/joim.12316 25285747

[6] Updated ESC (2011). Guidelines for managing patients with suspected non-ST-elevation acute coronary syndromes. Eur Heart J.

[7] Thygesen K, Alpert JS, White HD (2007). Universal definition of myocardial infarction. J Am Coll Cardiol.

[8] Borna C, Thelin J, Ohlin B, Erlinge D, Ekelund U. High-sensitivity troponin T as a diagnostic tool for acute coronary syndrome in the real world: an observational study. Eur J Emerg Med. 2013. Epub 2013/06/12. 10.1097/MEJ.0b013e328362a71b 23751287

[9] Bahrmann P, Heppner HJ, Christ M, Bertsch T, Sieber CC. Early detection of Non-ST-Elevation Myocardial Infarction in geriatric patients by a new high-sensitive cardiac Troponin T assay. Aging Clin Exp Res. 2011. Epub 2011/09/29. 10.3275/7927 21952408

[10] Chenevier-Gobeaux C, Meune C, Freund Y, Wahbi K, Claessens YE, Doumenc B, et al. Influence of Age and Renal Function on High-Sensitivity Cardiac Troponin T Diagnostic Accuracy for the Diagnosis of Acute Myocardial Infarction. The American journal of cardiology: Claessens YE; 2013. Epub 2013/04/02. 10.1016/j.amjcard.2013.02.024 23540652

[11] Normann J, Mueller M, Biener M, Vafaie M, Katus HA, Giannitsis E (2012). Effect of older age on diagnostic and prognostic performance of high-sensitivity troponin T in patients presenting to an emergency department. Am Heart J.

[12] Reiter M, Twerenbold R, Reichlin T, Haaf P, Peter F, Meissner J (2011). Early diagnosis of acute myocardial infarction in the elderly using more sensitive cardiac troponin assays. Eur Heart J.

[13] Gore MO, Seliger SL, Defilippi CR, Nambi V, Christenson RH, Hashim IA (2014). Age- and sex-dependent upper reference limits for the high-sensitivity cardiac troponin T assay. J Am Coll Cardiol.

[14] Thygesen K, Mair J, Giannitsis E, Mueller C, Lindahl B, Blankenberg S (2012). How to use high-sensitivity cardiac troponins in acute cardiac care. Eur Heart J.

[15] Kline JA, Johnson CL, Pollack CV, Diercks DB, Hollander JE, Newgard CD (2005). Pretest probability assessment derived from attribute matching. BMC Med Inform Decis Mak.

[16] Carro A, Kaski JC (2011). Myocardial infarction in the elderly. Aging Dis.

[17] Hamm CW, Bassand JP, Agewall S, Bax J, Boersma E, Bueno H (2011). ESC Guidelines for the management of acute coronary syndromes in patients presenting without persistent ST-segment elevation: The Task Force for the management of acute coronary syndromes (ACS) in patients presenting without persistent ST-segment elevation of the European Society of Cardiology (ESC). Eur Heart J.

[18] Sethi A, Bajaj A, Malhotra G, Arora RR, Khosla S (2014). Diagnostic accuracy of sensitive or high-sensitive troponin on presentation for myocardial infarction: a meta-analysis and systematic review. Vasc Health Risk Manag.

[19] Bahrmann P, Christ M, Bahrmann A, Rittger H, Heppner HJ, Achenbach S (2013). A 3-h diagnostic algorithm for non-ST-elevation myocardial infarction using high-sensitivity cardiac troponin T in unselected older patients presenting to the emergency department. J Am Med Dir Assoc.

[20] Olivieri F, Galeazzi R, Giavarina D, Testa R, Abbatecola AM, Ceka A (2012). Aged-related increase of high sensitive Troponin T and its implication in acute myocardial infarction diagnosis of elderly patients. Mech Ageing Dev.

[21] Frankenstein L, Wu AH, Hallermayer K, Wians FH, Giannitsis E, Katus HA (2011). Biological variation and reference change value of high-sensitivity troponin T in healthy individuals during short and intermediate follow-up periods. Clin Chem.

[22] Elf JL, Strandberg K, Svensson PJ (2010). The diagnostic performance of APC-PCI complex determination compared to D-dimer in the diagnosis of deep vein thrombosis. J Thromb Thrombolysis.

[23] Mueller M, Biener M, Vafaie M, Doerr S, Keller T, Blankenberg S (2012). Absolute and relative kinetic changes of high-sensitivity cardiac troponin T in acute coronary syndrome and in patients with increased troponin in the absence of acute coronary syndrome. Clin Chem.

[24] Reichlin T, Irfan A, Twerenbold R, Reiter M, Hochholzer W, Burkhalter H (2011). Utility of absolute and relative changes in cardiac troponin concentrations in the early diagnosis of acute myocardial infarction. Circulation.

[25] Reichlin T, Schindler C, Drexler B, Twerenbold R, Reiter M, Zellweger C (2012). One-hour rule-out and rule-in of acute myocardial infarction using high-sensitivity cardiac troponin T. Arch Intern Med.

[26] Katus HA, Remppis A, Scheffold T, Diederich KW, Kuebler W (1991). Intracellular compartmentation of cardiac troponin T and its release kinetics in patients with reperfused and nonreperfused myocardial infarction. Am J Cardiol.

[27] Rains MG, Laney CA, Bailey AL, Campbell CL (2014). Biomarkers of acute myocardial infarction in the elderly: troponin and beyond. Clin Interv Aging.

